# The limits of *p*-values for biological data mining

**DOI:** 10.1186/1756-0381-6-10

**Published:** 2013-05-11

**Authors:** James D Malley, Abhijit Dasgupta, Jason H Moore

**Affiliations:** 1 Center for Information Technology, The National Institutes of Health, Bethesda, MD, USA; 2 The National Institute of Arthritis and Musculoskeletal and Skin Diseases, The National Institutes of Health, Bethesda, MD, USA; 3 Departments of Genetics and Community and Family Medicine, Institute for Quantitative Biomedical Sciences, Lebanon, USA; 4 The Geisel School of Medicine, Dartmouth College, One Medical Center Dr, Lebanon, NH 03756, USA

## 

Use of *p*-values is widespread in the sciences, especially so in biomedical research, and also underlies several analytic approaches in data mining. Its original intent is simple enough, but its application and interpretation are far from simple. If data is collected to evaluate an idea, a hypothesis, then accepting the idea, when it is true, is a good thing, and rejecting the idea when it is not true, is also good. Two errors in reasoning from the data then can occur: a true idea is rejected (making a Type I error), or a false idea is accepted (Type II error).

However, simple criticisms and essential distinctions are immediate: (1) The *p*-value is not a probability of an idea being true; such a more evolved statement requires using Bayes theorem—at least—and a different frame for inference; (2) Just stating the result of a statistical test as a *p*-value is nearly uninformative, as a statistically significant outcome may have no practical biological importance; and continuing (3) The size of the departure from the proposed true idea, the effect size, could be quite small in the subject matter context; (4) The statistical method chosen for making a *p*-value declaration could be doubtful, or inappropriate (i.e. wrong); (5) The consequences of reasoning forward from a declared *p*-value has uneven consequences: so-called false positive and false negatives are rampant and often hard to reckon with in many biomedical testing environments (e.g. mammograms); all of which to say is (6) The utility or cost of false positives and false negatives is unexamined in simple *p*-value declarations.

All the above is well-known in the statistical community and much-studied over many years [1,2]. More recent problems with *p*-values include: (1) Correction for multiple testing, over hundreds, thousands or even millions of tests, using methods such as Bonferroni or False Discovery Rate (FDR). This occurs often in genomics and data mining and the corrections, or adjustments are often scientifically ungrounded and assume the universal null hypothesis that all findings are due to chance [3]. The central problem is that such testing assumes the separate *p*-values are in effect, independent agents, and the power to detect biological associations from one gene or genetic variant to the next are sent to zero. Introducing biologically realistic entanglements and higher order correlations across genomic sites and events is deeply problematic and nearly impossible to get right; (2) Another problem is that the reported *p*-values of such tests of association are weirdly at odds with current basic science. Consider, for example, quantum mechanics that is the single most experimentally well-validated understanding of basic physics ever proposed in the history of science. Despite its scientific rigor, quantum mechanics is accurate only to about eight or nine significant digits. However, it is not uncommon for researchers to report *p*-values of less than 10^-40^. Such assertions are reporting experimental testing outcomes more accurate than quantum mechanics, comparable to making declarations for events rarer than the decay rate of the proton.

Further, such small *p*-values cannot be justified by randomization or statistically grounded arguments given the relatively small sample sizes in play. They only announce a blind faith in the validity of an assumed distribution (like the chi-squared) for parsing an observed test outcome far into its tail.

A problem closely related to the strict reliance on *p*-values—and the two kinds of errors, the false negatives and positives—is the wide use of Receiver Operator Curves (ROC) curves. This scheme arose in the 1940s for testing the performance of a radio receiver and for that kind of device good reception across an entire bandwidth makes sense. So the device needs to have low reception error and high rejection of noise at many frequencies. For a medical test this usually makes no sense: The researcher makes a practical and scientific decision about applying the test (setting the threshold), and proceeds to use the test accordingly on the next patient. But the patient is not a radio under test. Next, the area under the ROC curve, the AUC value, is thought useful and often reported as determinative. However, it is easy to construct simple and plausible examples where the AUC estimate is unstable with multiple test outcomes all having AUC exactly equal to 1, and all being distinct in terms of inference.

So, given all the problems above, what good purpose is served, or could be served by *p*-values? This can be resolved by bringing the focus back to the scientific, data mining questions: What are the hypotheses of interest (are there different ways to frame the analysis)? Are the hypotheses under study related in some way (independent, not independent)? What are the costs of drawing the wrong conclusion (what at the underlying risks, estimated effect sizes)? Beyond *p*-values, FDR, ROC, and AUC, are there more efficient uses of the same data? What is truly predictive rather than being merely significant? This last question is, indeed, the single most critical and drives an informed and grounded response to all the others. We will explore these entangled issues in future editorials.
